# Study on the Solidification Effect of Dredger Fill by Microbial-Induced Calcium Precipitation (MICP)

**DOI:** 10.3390/ma15227891

**Published:** 2022-11-08

**Authors:** Jun Li, Lijun Tian, Yan Xu, Zefeng Tian, Zhendong Zhang

**Affiliations:** 1 School of Civil Engineering, Liaoning Technical University, Fuxin 123000, China; 2 Liaoning Key Laboratory of Mine Subsidence Disaster Prevention and Control, Liaoning Technical University, Fuxin 123000, China; 3 School of Civil Engineering, Shenyang Jianzhu University, Shenyang 110168, China; 4 Heguang Juneng (Liaoning) Environmental Protection Technology Co., Ltd., Fuxin 123000, China

**Keywords:** dredger fill, MICP, permeability, microstructure, reinforcement mechanism, urease activity

## Abstract

This paper puts forward a new soft soil reinforcement technology—microbial-induced calcite precipitation (MICP) technology—which considers the problem of dredger fill soft-soil reinforcement in Dalian Taiping Bay. In this paper, the calcium carbonate content (CCC) and unconfined compressive strength (UCS) of microbial solidified dredger fill (MSDF) samples were determined using laboratory experiments. The microstructure and chemical composition of MSDF samples were studied by SEM–EDS and XRD. The failure and reinforcement mechanism of MSDF under different experimental conditions (ambient temperature, cementation solution concentration, and clay content) were investigated. The results showed that there was a certain residual strength after the peak strength of MSDF. With the increase of ambient temperature, the number of microorganisms increased, but the activities of urease, CCC, and UCS decreased. The UCS and CCC increased with the increase of cementation solution concentration, while they first increased and then decreased with the increase of clay content. The clay content enhanced the compactness of MSDF samples but reduced the soil permeability and weakened the mineralization. There were significant differences in the morphology of microbial-induced precipitation caused by different concentrations of cementation solution.

## 1. Introduction

The shortage of land resources in coastal areas has limited the development of engineering construction. Sea sand reclamation can effectively alleviate this problem, but the foundation formed by hydraulic reclamation soft soil faces the problem of having insufficient bearing capacity. Dredger fill refers to the sedimentary soil formed by dredging the sludge that has accumulated at the bottom of the river for a long time, which is then transported to its intended destination using hydraulic dredgers. It is widely used in land reclamation projects because of its convenient properties for construction purposes, compliance with environmental protection measures, and availability [1]. The traditional soft soil foundation treatment methods of dredger fill face problems such as high cost, poor uniformity, considerable disturbance to the ecological environment, and some potential safety hazards [2,3,4]. Therefore, it is necessary to carry out further scientific research on the application prospect of dredger fill foundation stability and reinforcement in land reclamation.

When considering dredger fill in the field of construction engineering, the traditional vacuum preloading method is faced with construction difficulties on site [5]. Liu et al. [6,7] proposed an improved multiple vacuum preloading method for strengthening the soft soil foundation of dredger fill in the Tianjin Lingang Industrial Zone, China. Wang et al. [8] put forward a two-stage vacuum preloading method and focused on the feasibility and effectiveness of dredged marine clay filler for land reclamation in engineering construction. Hu et al. [9] put forward the theory of deformation and bearing capacity based on the characteristics of discontinuous deformation and low strength of dredger fill and studied the deformation and failure process of subgrade dredger fill. Wang et al. [10] studied the geological conditions, material properties and mechanical behavior of the coral reef reclamation area in the South China Sea in combination with the characteristics of uneven coral particle size distribution, irregular particle shape, broken particles, high porosity, and poor uniformity. With the development of nondestructive testing technology, Yu et al. [11] used X-ray computed tomography (CT) to observe the internal structure and investigated the physical properties and compression characteristics of dredger fill. Tang et al. [12] studied the influence of organic matter and soil pH value on the consolidation degree of viscous dredger fill through a series of small-scale vacuum preloading tests and evaluated the effect of vacuum preloading consolidation through scanning electron microscopy.

**Figure 1:**
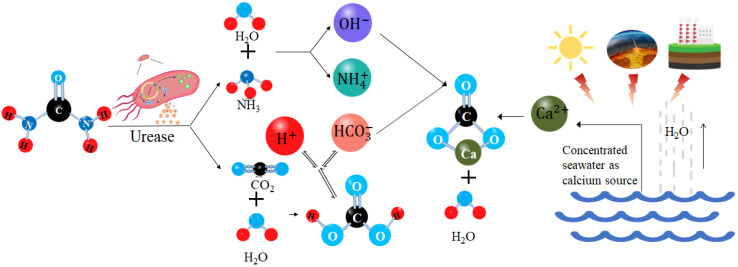
Principle of MICP.

Microbial-induced calcite precipitation (MICP) is a technology that uses microbial urease to induce calcite mineralization and it is widely used in geotechnical engineering [13]. Figure 1 shows the schematic diagram of MICP. Urea in nature is catalyzed by microbial urease to form carbonate ions (CO_3_^2−^) through a series of reactions. When carbonate combines with calcium ions in soluble salts, gel-like white calcium carbonate crystals precipitate. Liang Cheng et al. [14] studied the in situ soil reinforcement technology of MICP asserting the advantages of inorganic minerals in the solidification of dredger fill soft soil making up for the poor cohesion of traditional reinforcement methods. Chu et al. [15] studied the effects of centrifuged bacterial suspensions, diluted bacterial suspensions, and bacteria-free culture broths of *Bacillus Pasteurella* on microbial-induced calcite precipitation in sandy soils. Cheng et al. [16] studied the calcium carbonate content in sand samples after MICP treatment at different temperatures, and the results showed that the calcium carbonate content of sand samples treated at high temperature was three times that of those at room temperature, but the calcium carbonate crystal generated at room temperature had a large diameter and formed more effective cementation. Zamarreno et al. [17] found that with an increase of temperature, the amount of carbonate precipitation in the liquid medium environment also increased and affected the quality and morphology of carbonate precipitation. Cacchio et al. [18] studied the ability of different strains to produce calcite precipitation and found that the degree of microorganism mineralization and the type of calcium carbonate crystal were related to the culture temperature of the bacteria. Mahawish et al. [19] studied the influence of aggregate size distribution on the effect of biological cementation by adding fine aggregate to coarse aggregate. Muazza et al. [20] studied the self-healing process of MICP in concrete using a native strain of adaptive *Bacillus cereus* isolated from Qatari soil, which had the advantage of withstanding and executing MICP in an environment of 45–50 °C and 80%–100% relative humidity. Burbank et al. [21] studied the mechanical properties of soil solidified using indigenous microorganisms by injecting grouting into the soil to activate the native urease microorganisms in the soil and cement the soil. Gomez et al. [22,23] and Graddy et al. [24] compared and studied the effect of solidification of sandy soil using native microorganisms and artificially cultured microorganisms and analyzed the difference of urease activity between native microorganisms and cultured microorganisms. Nafisi et al. [25] carried out triaxial drainage tests and verified the nonlinear failure envelope of MICP treated sand by comparing the shear stress and normal stress at the time of failure with the stress predicted by the nonlinear failure envelope. Gowthaman et al. [26,27] studied the application of MICP in the field of slope protection. Their results show that slope instability can be prevented by microbial cementation. Sharma et al. [28] studied the durability of bonded sand and the influence of weathering on liquefaction resistance. The research results of Van Paassen et al. [29], Whiffin et al. [30] and Sharma et al. [31] provide the for possibility of field production and field application.

Although previous scientific research has been carried out on the mechanical properties and mesofabric of microorganism mineralization in solidified soil, research on the differences in the microorganism mineralization effect and the mechanism caused by environmental factors and cement solution concentration is very rare. This paper focuses on the factors affecting the UCS of microorganism solidified dredger fill samples. The mechanical properties of the solidified dredger fill soft-soil material are studied by macro testing the density, UCS, CCC, and permeability of the solidified dredger fill, The microscopic process of calcium carbonate precipitation induced by *Sporosarcina pasteurii* in nutrient solution, the solidification and bonding process of soft soil particles, and the microstructure and mineral composition of solidified soil were analyzed by optical and electronic microscopy, and X-ray diffraction. The research results provide scientific guidance for the application of microorganism mineralization technology in the field of dredger fill soft-soil solidification.

## 2. Materials and Methods

### 2.1. Soil

**Figure 2:**
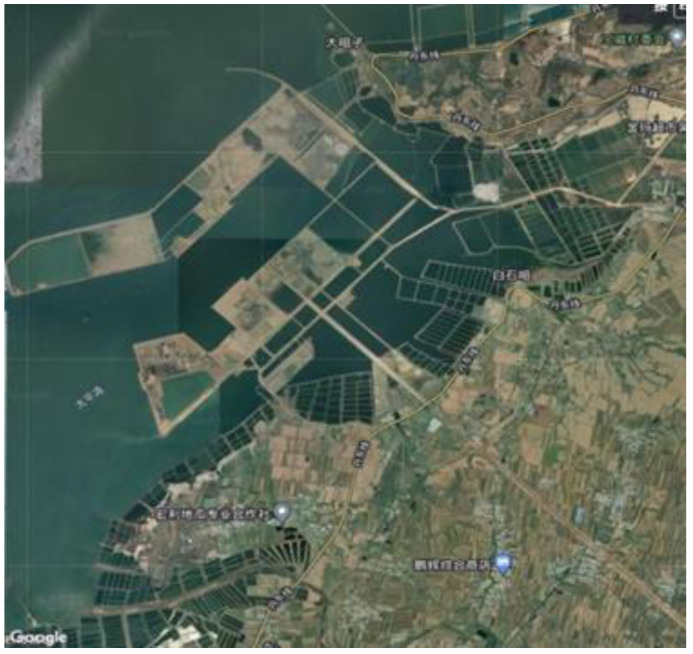
Construction progress of Taipingwan port area.

**Figure 3:**
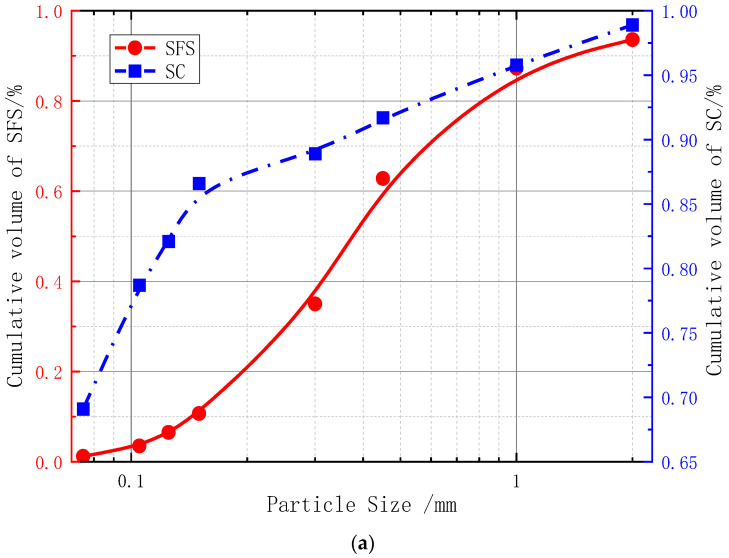

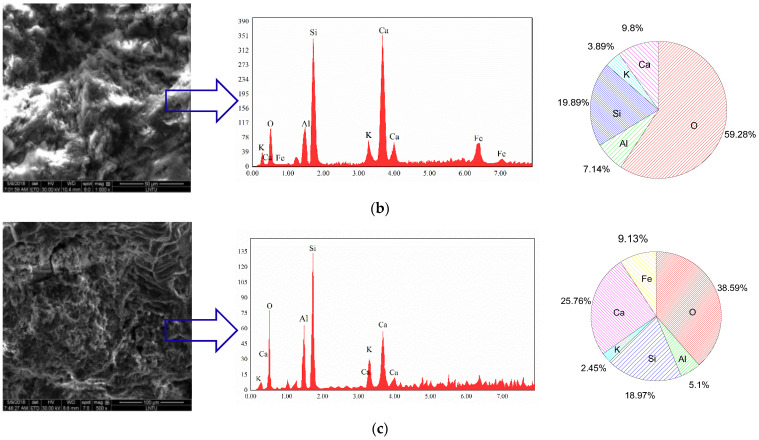
Particle size distribution and chemical components. (**a**) Particle size distribution. (**b**) Microscopic morphology and chemical composition of SFS. (**c**) Microscopic morphology and chemical composition of SC.

The dredger fill used in the test was from the Taiping Bay port area of Dalian, China (as shown in Figure 2; 39°52′~40°15′ N, 121°32′~122°06′ E) and was mainly composed of silty fine sand (SFS) and silty clay (SC). The SFS was mainly composed of Sinian Yongning sandstone, granite, other quartz and feldspar, and a small amount of shell debris, while the SC was mainly composed of seabed silt and a very small amount of Sinian Yongning sandstone quartz sand. The basic physical and mechanical parameters of the SFS and the SC tested according to the relevant Chinese experimental operating specifications [32] are shown in Table 1. The particle size distribution was determined by the pyknometer method and the microscopic morphology and chemical composition were tested using the SEM–EDS method. The test results are shown in Figure 3a–c.

Figure 3a demonstrates that the particle size distribution of the SFS was more uniform than that of SC. Figure 3b,c shows that the main components of the SFS were calcium silicate (C_2_S, C_3_S) and calcium aluminate 3CaO·Al_2_O_3_, while the composition of the SC was relatively complex, in addition to calcium silicate and calcium aluminate, it also contained a small amount of tetracalcium aluminate (C_4_AF).

### 2.2. Urease Bacteria and Cementation Solution

#### 2.2.1. Bacterial Activation

**Figure 4:**
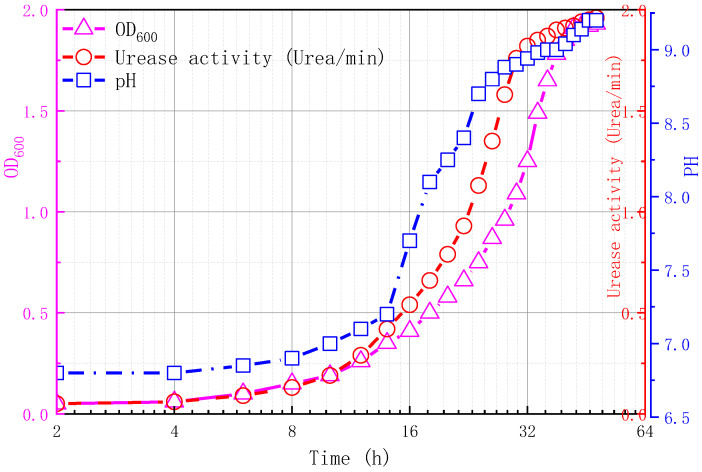
Changes of OD_600_, urease activity, and pH.

First, casein peptone (15 g), soybean peptone (5 g), sodium chloride (5 g), and urea (20 g) were added to distilled water (1000 mL) to prepare the culture medium. The prepared culture medium was fully stirred with a glass rod, the pH was adjusted to nine with 1 mol/L NaOH solution, distributed into a conical flask, and sterilized at 121 °C for 30 min. Then *Sporosarcina pasteurii* (BNCC337394; Beina Chuanglian Biotechnology Co., Ltd., Beijing, China) was used for expanded culture to prepare a bacterial solution. The spectrophotometry of the bacterial solution at the wavelength of 600 nm (OD_600_) was tested by a 722s visible spectrophotometer and the conductivity was measured every 30 min for 48 h to determine the urease activity. The OD_600_, urease activity, and pH value of the mixture are shown in Figure 4 where, 0–8 h is the adaptation stage of bacteria, during which the number of bacteria increases slowly; 8–30 h is the logarithmic growth stage of bacteria, during which bacteria grow rapidly; 30–48 h is the equilibrium stage, and the number of bacteria is relatively stable. After 48 h, the bacteria die and the number of bacteria decrease. In this paper, the stable bacterial solution was taken for subsequent testing.

#### 2.2.2. Culture Medium and Cementation Solution

The cementation solution is a mixture of urea and calcium chloride with a volume ratio of 1:1, in which urea provides the nitrogen source and energy for the growth of microorganisms and calcium chloride provides calcium ion for the reinforcement of dredger fill soft soil. According to the biochemical reaction mechanism of MICP, the CCC increased with the increase of the cementation solution concentration [33]. Therefore, four levels of concentration (0.5 mol/L, 1.0 mol/L, 1.5 mol/L, and 2.0 mol/L) were selected to study the influence of cementation solution concentration on the MICP reinforcement effect.

### 2.3. Apparatus and Sample Preparation

In the MICP process, the influence of precipitation is affected by ambient temperature and cementation solution concentration, and the addition of clay can improve the particle gradation. In this paper, the physical and mechanical properties of SFC and CC were studied by setting different ambient temperatures, cementing solution concentrations and clay content.

**Figure 5:**
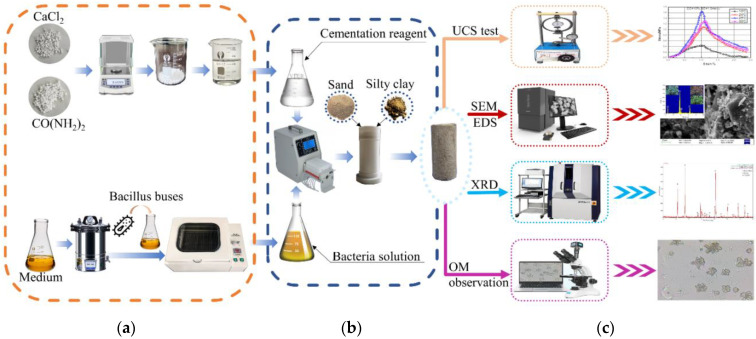
Sample preparation and testing. (**a**) Solution preparation, (**b**) Sample preparation, (**c**) Test.

As shown in Figure 5b, the mixture of SFS and SC was placed into a cylindrical mold with a diameter of 50 mm and a height of 100 mm in five (20 mm thick) layers and compacted by vibration. First, a rubber dropper was used to take up 10 mL of bacterial solution and slowly drip it into the soil. This was left for 10 min or until there was no exudate on the surface of the soil sample and the bacterial solution stopped dripping. Then, 10 mL of cementation solution and 10 mL of urea solution was slowly injected into the soil column mold with a peristaltic pump. It was then left for a further 10 min until there was no exudate on the surface of the MSDF sample, so as to complete the pregrouting experiment. The above steps were repeated and the curing time of each group of MSDF samples was eight days. The curing reaction was carried out under constant temperature and humidity conditions for 48 h. The specimen grouting and preparation scheme is shown in Table 2.

### 2.4. Test Scheme

#### 2.4.1. Unconfined Compressive Strength (UCS)

According to the regulations of the Chinese geotechnical test standard (GBT 50123-2019), the unconfined compressive strength (UCS) of the MSDF samples were tested by a strain-controlled unconfined manometer. The loading plate was raised for testing, every 0.5% strain (0.4 mm) was read, and the test was completed within 10 min. When the peak value appeared in the loading curve, 3–5% of the strain was further applied after reaching the peak stress. When the loading curve reached its peak, 3–5% of the strain was further applied before stopping the test, and when the loading curve had no peak, the loading strain reached 20%, and the axial stress on the specimen was calculated according to Equation (1).

(1) $$\sigma =\frac{C\times R}{A_{a}}\times 10$$

where, σ is the axial stress (kPa); C is the calibration coefficient of dynamometer (N/0.01 mm); *R* is the reading of dynamometer (0.01 mm); and *A_a_* is the area of the specimen in shear (cm^2^).

#### 2.4.2. Content of Calcium Carbonate (CCC)

The crushed samples after unconfined compressive strength test were collected and stored in sealed bags, and the content of calcium carbonate (CCC) produced in MSDF samples was determined by the acid pickling method. The pickling method was to treat MSDF samples (approximately 5 g) with an excess hydrochloric acid solution with a concentration of 1 mol/L, filter them with filter paper, rinse them with ultrapure water several times, dry them, and measure the mass reduction of the samples, that is, CCC.

The soil used as dredger fill soft soils contained some seashells, whose main component is calcium carbonate and dissolves under the application of acid, which interfered with the mass measurement of the calcium carbonate generated by MICP. Therefore, before the CCC test, the uncured dredger fill soft soil of the same quality as MSDF was selected for acid washing treatment to obtain the calcium carbonate content of the uncured dredger fill soft soil itself.

#### 2.4.3. Permeability

The permeability of MSDF samples was measured by the variable head method. The vertical variable head pipe was connected through the rubber pipe (*φ* = 8 mm) connected to the Buchner funnel at the bottom of the curing device, and the air tightness of the curing device was checked. When the water head rose to a predetermined height, the water inlet pipe clamp opened to make water flow from the bottom of the MSDF sample and overflow from the top. At this time, the initial water head height *H*_1_, the end water head *H*_2_, the time changes Δ*t* in the variable water head pipe, and the water temperature at the water outlet were measured. The water level height in the head pipe was changed, then the change of water head was measured and the time when the water level became stable was recorded. This test was repeated five to six times. The variable head permeability coefficient was calculated according to Equation (2) [34].

(2) $$k=2.3\frac{aL}{A\Delta t}\mathrm{lg}\left(\frac{H_{1}}{H_{2}}\right)$$

where, *a* is the cross-sectional area of the variable head pipe (cm^2^), *L* is the height of soil column (cm), Δ*t* is the time interval (s), and *A* is the cross-sectional area of the sample (cm^2^).

#### 2.4.4. Void Ratio

The void ratio of the MSDF sample was measured using the drying method. The MSDF sample was saturated, and the wet weight of the sample was measured after 24 h and denoted as *M_W_*. Then the MSDF samples were dried to constant weight in an oven at 60 °C, and the dry weight was recorded as *M_d_*. The void ratio of the sample can be calculated according to Equation (3).

(3) $$n=\frac{M_{w}-M_{d}}{\rho_{w}V}\times 100\%$$

where, $\rho_{w}$ is water dendity, V is sample volume

Sample preparation and the testing process are shown in Figure 5.

## 3. Results and Discussion

### 3.1. Relationship between UCS and CCC of MSDF

#### 3.1.1. Stress–Strain Characteristics of MSDF

**Figure 6:**
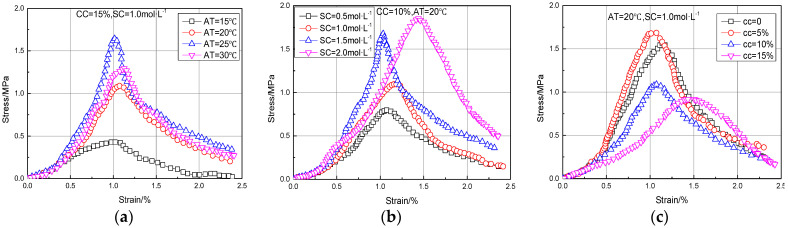
Test results under different conditions of AT, SC, and CC. (**a**) Group A. (**b**) Group B. (**c**) Group C.

**Figure 7:**
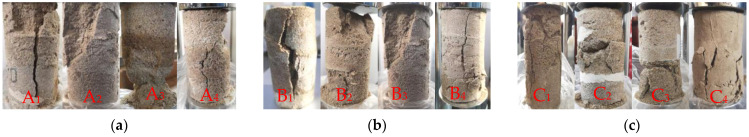
Failure modes under different AT, SC, and CC conditions. (**a**) Group A. (**b**) Group B. (**c**) Group C.

Figure 6 and Figure 7 show the stress–strain curve and failure mode of MSDF samples after the UCS test under different ambient temperatures, cementation solution concentrations, and clay content, respectively.

Figure 6a–c show that the stress–strain curve can generally be divided into four stages. The stress–strain curve under different conditions shows the characteristic of strain softening, with an obvious peak stress, which is the UCS of MSDF samples.

Figure 7a shows that a top-down through crack formed in the middle of the section of sample A1, an oblique crack failure occurred in sample A2, sample A3 remained semi complete, the end in contact with the loading plate was partially disassembled, and sample A4 remained relatively complete. Figure 7b shows that sample B1 demonstrated overall shear failure, while sample B2 demonstrated crushing failure; the weak cementation area of samples B3 and B4 was significantly reduced, and the samples remained relatively intact, indicating that the strength of SFS and SC had been significantly improved. Figure 7c shows that the C1 sample had a top-down through crack, C2 and C3 samples had skin peeling in the middle but they were not completely damaged, C4 sample had a crack on the surface and protruded, with the continuous development of the crack, and the sample had local crack damage.

The stress–strain curves under different conditions of AT, SC, and CC were consistent and similar. The stress of the sample increased with the increase of strain at first, and then decreased with the increase of strain after reaching the peak value. Liu et al. [35] also reached the same conclusion. At different ambient temperatures, the strain of MSDF specimen was about 1%, and the peak stress at 25 °C was the largest. With the increase of cementation solution concentration, the peak stress increased continuously, and when the concentration was 2 mol/L the strain value obviously increased, which was around 1.5%. When the content of clay particles was 5%, the corresponding peak stress was the largest and the strain was reduced, which indicates that the content of clay particles changed the bonding degree of particles.

#### 3.1.2. Relationship between UCS and CCC

**Figure 8:**
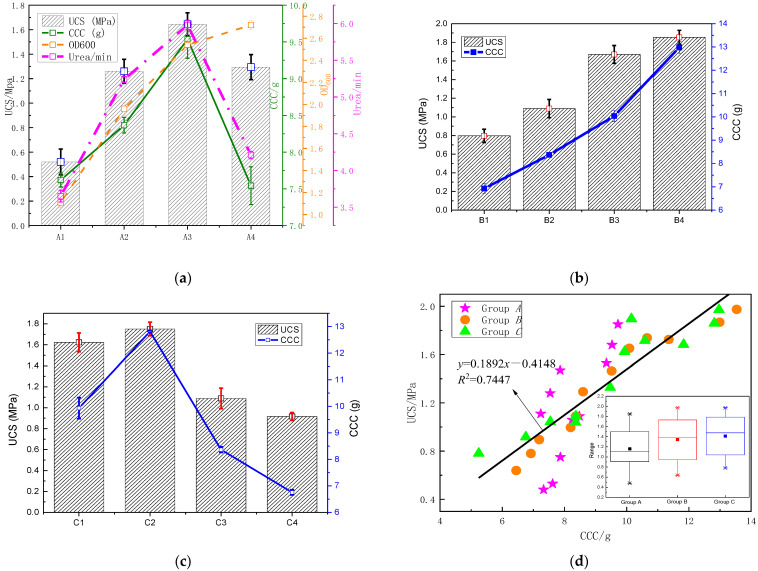
Relationship between UCS and CCC of MSDF. (**a**) Group A, (**b**) Group B, (**c**) Group C, (**d**) Relationship between UCS and CCC.

Figure 8a shows that when the ambient temperature reached 25 °C, the UCS of MSDF reached the maximum of 1.64 MPa, and the corresponding (CCC) was 9.54 g. While when the temperature exceeded 25 °C, even though the density of bacteria (OD_600_) increased with the increase of temperature, the urease activity began to decrease, indicating that the UCS and CCC of MSDF samples were directly related to urease activity but not absolutely related to bacterial OD_600_. This was mainly because when the temperature exceeded 25 °C, although the proliferation of microorganisms increased rapidly, the nutrients and space occupancy of urease bacteria per unit volume decreased, forming a competitive relationship with each other and resulting in the decline of urease activity.

Figure 8b shows that the CCC and the UCS increased with the increase of the cementation solution concentration. When the cementation solution concentration reached 2.0 mol/L, the CCC reached 12.98 g and the UCS reached 1.85 MPa, and both the CCC and the UCS reached the maximum. This is because the high concentration of cementation solution can provide a sufficient source of calcium for mineralization. With the microbial-induced mineralization, a large amount of calcite was generated, which had cementation and gradually increased the UCS of MSDF.

Figure 8c shows the CCC and the UCS first increased and then decreased with the increase of clay content (CC). When the CC reached 5% of the total weight of the MSDF samples, both the CCC and the UCS reached the maximum, which were 12.81 g and 1.75 MPa, respectively. This was mainly because when the CC was less than 5%, the addition of clay could improve the particle gradation of dredger fill soft soil, but if the content of clay exceeded 5%, it blocked the pore channel between dredger fill soft-soil particles, reducing the permeability and mineralization effect of the MSDF samples.

The above discussion demonstrates that under the experimental conditions of this paper, the influence of temperature on MICP was related to urease activity, and the optimum temperature was 25 °C, at which the UCS value reached the maximum. This further confirms the hypothesis that the crystal formation factors mentioned in reference [16] may be related to urease activity. In the process of cementation solution concentration, liquid will scour the sample. When the scouring rate is higher than the binding rate of calcium carbonate, the formed calcium carbonate gel will be washed away by the water flow. When the concentration was less than 2 mol/L, the scouring and binding could not reach the equilibrium state, so the UCS value was low. CC mainly changes the particle gradation of dredger fill. When CC was 5%, the gradation effect was the best, and the UCS value of the sample was the highest. These results can provide practical guidance value for engineering.

Figure 8d shows that the relationship between UCS and CCC of MSDF under different experimental conditions could be obtained by linear regression method as shown in Equation (4).

(4) y=0.1892x−0.4148

The regression results of Equation (4) show that the correlation coefficient can reach 0.7447, indicating that the UCS and the CCC have good linear correlation relationship. This is consistent with the research results of Zhao et al. [36] where UCS was positively correlated with CCC. However, on the basis of previous studies, the regression equation and correlation coefficient between UCS and CCC were obtained in this paper. 

### 3.2. Influences of CC on Void Ratio, Permeability, and CCC

**Figure 9:**
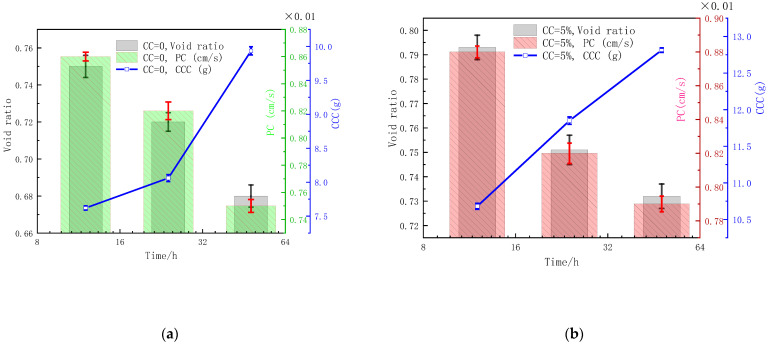

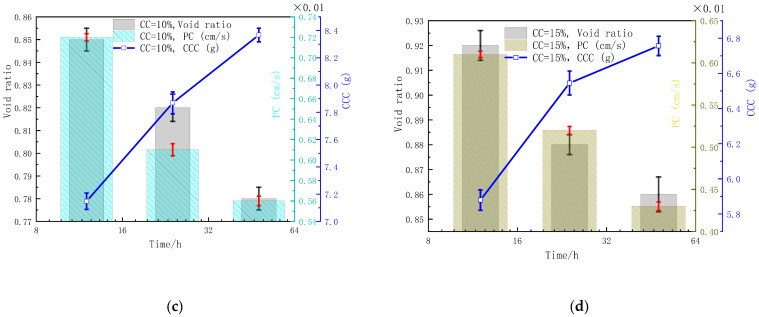
Influences of CC on the void ratio, permeability, and CCC. (**a**) CC = 0. (**b**) CC = 5%. (**c**) CC = 10%. (**d**) CC = 15%.

Figure 9 shows the evolution law of the void ratio, permeability, and CCC of MSDF samples with different clay contents at different stages.

As shown in Figure 9, from 12 h to 48 h, the void ratio of MSDF samples with a clay content of 0, 5%, 10%, and 15% decreased by 9.33%, 7.69%, 8.23%, and 6.52%, respectively; the permeability decreased by 12.79%, 10.23%, 22.22%, and 29.51%, respectively; and the generated calcium carbonate increased by 30.35%, 19.98%, 17.05%, and 14.93%, respectively. Table 3 gives the change rate of the void ratio, permeability, and CCC.

Table 3 shows that for the clay-free MSDF, the permeability decreased from 3.33 × 10^−5^ cm/S/h to 2.92 × 10^−5^ cm/S/h, while the formation rate of calcium carbonate increased from 3.67 × 10^−2^ g/h to 7.81 × 10^−2^ g/h, indicating that the adsorption of soil particles in bacteria was reduced and the amount of microorganism-induced mineralization was limited. When the reaction time exceeded 12 h, the induced calcium carbonate in the early stage provided attachment sites for the bacteria of subsequent mineralization, and the mineralization efficiency was improved.

In the period of 12 h–24 h, when MSDF was mixed with 10% clay, the reduction rate of the void ratio reached the minimum of 2.5 × 10^−3^/h, and the reduction rate of permeability reached the maximum of 9.17 × 10^−5^ cm/s/h, while the formation rate of calcium carbonate gradually decreased with the increase of clay content.

In the period of 24 h–48 h, when clay was added as 10%, the void ratio reduction rate reached the minimum of 1.67 × 10^−3^/h, the reduction rate of permeability gradually increased with the increase of clay content, and the formation rate of calcium carbonate gradually decreased with the increase of clay content. This was mainly because the clay distributed between the DF particles and had strong adsorption, which hinders the movement of early bacterial solution and cementation solution, and the generated calcium carbonate hindered the penetration of subsequent bacterial solution and cementation solution, affecting the uniformity of mineralization in MSDF. Therefore, the formation rate of calcium carbonate in the later stage decreased.

From the above analysis, it can be concluded that MDSF with different CC content changes the void ratio and PC through the CCC generation rate. When CC was not contained, soil particles in the early stage adsorbed less bacteria and generated less CCC. After 12 h, CaCO_3_ generated in the early stage became the adsorption site of bacteria, which led to a high mineralization rate in the later stage. With the increase of CC content, CC was distributed among DF particles, which hindered the movement of bacteria liquid and cementation solution in the early stage, resulting in uneven infiltration and affecting the uniformity of mineralization in the later stage. Lin et al. [37] found that the distribution of CaCO3 will affect the permeability. This paper studied the effect of CaCO3 formation rate on the porosity and permeability.

The above research demonstrates that MICP technology can improve the strength of soil and reduce the permeability. Particularly, when the cementation solution concentration was 2 mol/L, the maximum stress obtained was 1.85 Mpa. The literature [38,39] indicate that the strength could meet the construction’s purpose which is important to improve the safety and stability of soil.

### 3.3. SEM–EDS and XRD Analysis

#### 3.3.1. SEM–EDS Analysis

**Figure 10:**
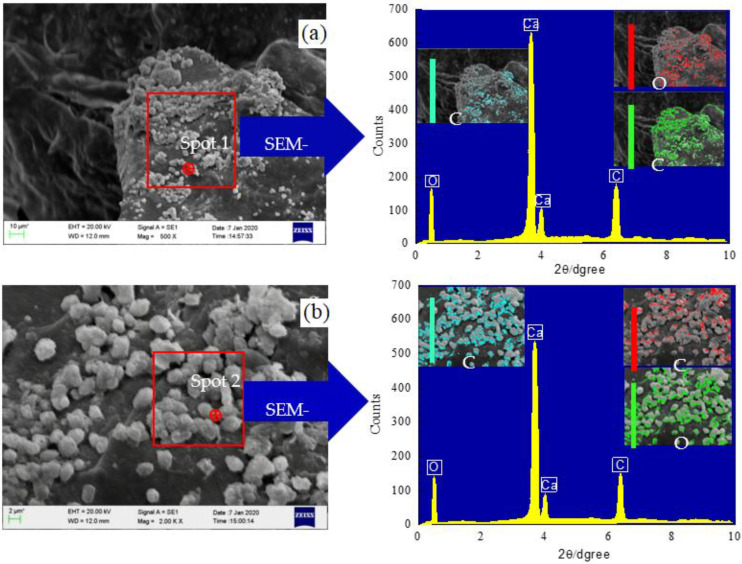
SEM–EDS analysis. (**a**) T = 15 ℃, (**b**) SC = 0.5 mol/L.

The SEM–EDS tests were performed with the Zeiss field emission microscopy (FEM) at an accelerating voltage of 15 kV. Figure 10a,b show the microstructure and chemical composition under the conditions of an ambient temperature of 15 °C and a cementation solution concentration of 0.5 mol/L, respectively.

**Figure 11:**
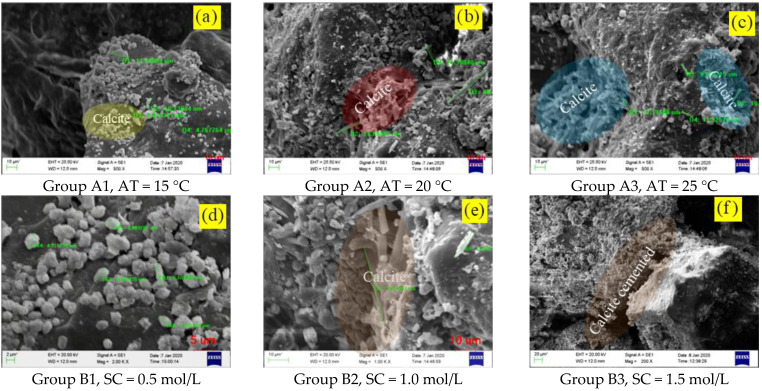

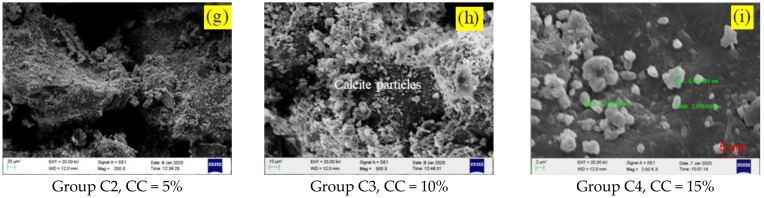
The presence of CaCO_3_ under different conditions.

According to the EDS test results of spot1 and spot2 in Figure 10a,b, the induced minerals distributed on the surface of dredger fill particles mainly composed of three chemical elements: C, O, and Ca, which are the main components of calcite crystals produced in the MICP process. The meso- and microstructure and spatial distribution of minerals induced by microorganisms under different conditions are shown in Figure 11a–i.

Figure 11a–c show the calcium carbonate induced by microorganisms cemented the scattered particles together. When the ambient temperature reached 15 °C (relatively low), due to the close relationship between microbial urease activity and ambient temperature, calcite crystals were dispersed on the particle surface. The minimum and maximum particle sizes of calcium carbonate were 4.77 μm and 17.20 μm, respectively. The microbial-induced minerals with uneven particle size limit the cementation effect of the calcium carbonate to the DF particles. When the temperature rose to 20 °C, the activity of urease increased.

Urea in the environment decomposed rapidly under the catalysis of urease, and the catalytic product combined rapidly with calcium ions to form an acicular calcite crystal with a maximum size of 48.49 μm, which could form firm cementation between loose DF particles. When the ambient temperature rose to 20 °C, the activity of urease increased, the urea in the environment decomposed rapidly under the catalysis of urease, and the catalytic product combined rapidly with calcium ions to form an acicular calcite crystal with a maximum size of 48.49 μm, which could form a strong bond between macroporous particles and loose DF particles. When the ambient temperature rose to 25 °C, as urease activity continued to increase, the decomposition rate of urea catalyzed through urease accelerates, resulting in a further increase in the number of mineral crystals, with the maximum crystal size reaching 19.96 μm.

Figure 11d–f show that when the concentration of cement liquid was 0.5 mol/L, the scouring effect of cement liquid on pores was stronger than the mineralization of calcium ions combined with carbonate, and there were less calcite crystals formed on the particle surface, which made it impossible to effectively build a complete dredger fill particle skeleton. When the concentration of cementation solution reached 1.0 mol/L, microorganisms induced calcium ions to form strip calcite densely distributing among particles with a maximum calcite particle size of 38.85 μm. When the concentration of cementation solution reached 1.5 mol/L, the microorganism-induced calcium carbonate crystals were evenly distributed among the particles, which played a good cementation role for the loose DF particles and formed a complete particle skeleton.

Figure 11g–i show that when the clay content reached 5%, the internal-induced minerals were relatively evenly distributed and abundant. When the clay content reached 10%, the microorganism-induced minerals began to distribute unevenly in MSDF, and the partial cementation between particles was incomplete. This is mainly because with the increase of clay content, the passing rate of bacterial solution and cementation solution in the sample decreased, resulting in uneven distribution in MSDF samples. When the clay content reached 15% of the total mass, the influence of microbial-induced mineralization was further weakened, calcium carbonate precipitation only occurred locally on DF particles, and the maximum calcite grain size was 5.84 μm. This further verifies the fact that the content of microbial-induced mineralization gradually decreased with the decrease of permeability.

#### 3.3.2. XRD Analysis

**Figure 12:**
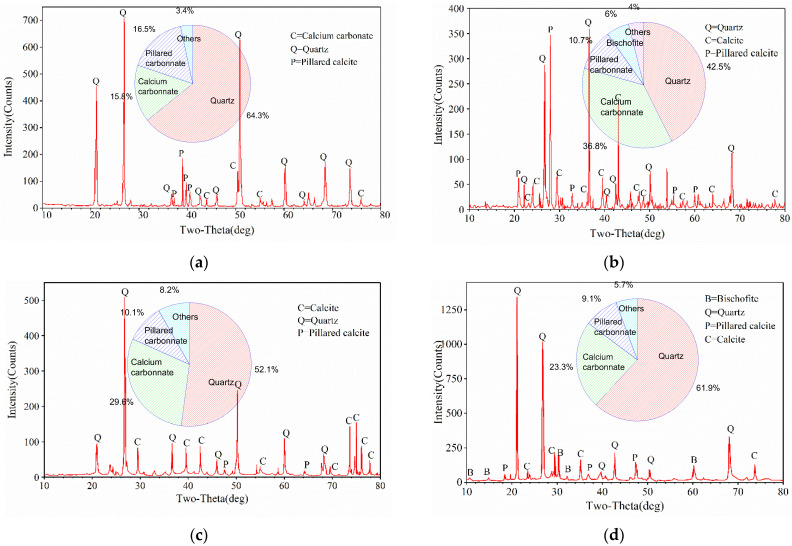
X-ray diffraction test results. (**a**) 0.5 mol/L. (**b**) 1.0 mol/L. (**c**) 1.5 mol/L. (**d**) 2.0 mol/L.

The X-ray diffraction tests were performed on MSDF samples with different cementation solution concentrations, and the data of diffraction peaks are obtained, including the position, peak height, FWHM, interplanar spacing and relative intensity. The mineral composition of the MSDF samples under different cementation solution concentration are shown in Figure 12a–d, respectively.

As shown in Figure 12a, the main mineral components of group B1 were quartz (${\mathrm{SiO}}_{2}$), calcium carbonate (${\mathrm{CaCO}}_{3}$), and pillared calcite (${\mathrm{Ca}}_{7}{\mathrm{Si}}_{6}O_{18}{\mathrm{CO}}_{3}{\left(H_{2}O\right)}_{2}$), which accounted for 64.3%, 15.8%, and 16.5%, respectively. As shown in Figure 12b, the main mineral components of group B2 were quartz, calcium carbonate, and pillared calcite, which accounted for 61.9%, 23.3%, and 9.1%, respectively. As shown in Figure 12c, the main mineral components of group B3 were quartz, calcium carbonate, and pillared calcite, which accounted for 52.1%, 29.6%, and 10.1%, respectively. As shown in Figure 12d, the main mineral components of group B4 were quartz, calcium carbonate, pillared calcite, and bischofite (${\mathrm{CaSi}}_{2}O_{5}\cdot 2H_{2}O$), which accounted for 42.5%, 36.8%, 10.7%, and 4%, respectively.

The XRD results show that in addition to calcium carbonate and pillared calcite, the microorganism-induced minerals also contained calcium silicate, which is a spherical, white, or colorless crystal, and is associated with calcite, dolomite, zeolite, and other minerals. The formation mechanism of calcium silicate is similar to that of pillared calcite, which is the product of hydration reactions such as calcium chloride and quartz. The new minerals detected by XRD diffraction included calcite, pillared calcite, and calcium silicate, in which, the calcium carbonate crystal was the only calcite phase in phase analysis, because calcite belongs to the most stable phase of calcium carbonate crystal in thermodynamics.

Through the observation and analysis of optical microscopy and SEM, the pillared calcite precipitated in a columnar shape from the surplus calcium chloride crystal in the induction reaction and the newly injected microorganism induced the mineralization reaction to form a stable crystal phase.

#### 3.3.3. Effect of Cementation Solution Concentration on Mineralization

**Figure 13:**
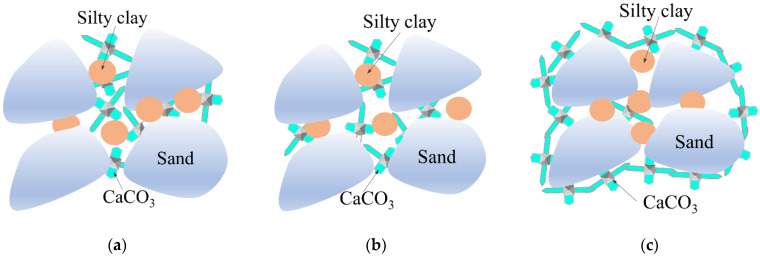
Differences of mineralization modes of different cementation solution concentrations. (**a**) Calcite precipitation. (**b**) Calcite cruciform cementation. (**c**) Calcite wrapped cementation.

As shown in Figure 13a–c, under the action of microbial-induced calcite precipitation, the cementation mode between DF particles can generally be divided into three types: calcite precipitation, calcite cruciform cementation, and calcite wrapped cementation. The urease produced by *Sporosarcina pasteurii* microorganisms is dispersed between DF and silty clay particles, which plays a positive role in enriching CaCO_3_ precipitation in a certain nutrient solution environment.

As shown in Figure 13a, when the concentration of the cementation solution was 0.5 mol/L, only a small amount of calcium carbonate precipitated and formed cruciform crystals deposited at the contact interface of the particles. This increased the roughness of the surface of the DF particles resulting in an increase of friction angle, thereby improving the shear strength. As shown in Figure 13b, when the concentration of the cementation solution increased to 1.0 mol/L, cruciferous crystals formed by calcium carbonate precipitation and crystallization adhered to the contact surface of the adjacent dispersed soil particles, the induced calcite crystals showed a cross shaped spatial network structure, and the dispersed DF particles were cemented one by one to form a complete spatial soil skeleton structure. As shown in Figure 13c, when the concentration of the cementation solution was 2.0 mol/L, a large amount of calcium carbonate gel was generated in the DF particle system, forming a complete spatial structure with DF particles wrapped in calcium carbonate. Therefore, under different cementing solution concentrations, the amount of calcium carbonate precipitation produced between DF particles was different, which ultimately led to different connection methods between soil particles, thus affecting the overall strength characteristics of MSDF to different degrees.

## 4. Conclusions

The influence of ambient temperature, cementation solution concentration, and clay content on the physical-mechanical properties of MSDF, meso- and microstructure, and chemical components were studied and the conclusions are as follows:

1. Under different conditions, the stress–strain curves of MSDF showed the characteristics of strain softening with obvious peak stress, which was the UCS value. The research showed that UCS had an obvious linear relationship with CCC, which can be expressed as y = 0.1892x − 0.4148.
2. The environmental temperature mainly affected urease activity, the concentration of cement solution mainly affected the total amount of calcium, and the content of clay mainly affected the particle size distribution, which led to the difference of microorganism-induced DF particle size. The optimum ore-forming conditions were 25 °C, a cementation solution concentration of 2 mol/L, and a 5% SC content.
3. The cementation mode between DF particles could generally be divided into three types: calcite precipitation, calcite cruciform cementation, and calcite wrapped cementation. The urease produced by *Sporosarcina pasteurii* microorganisms was dispersed between DF and silty clay particles, which played a positive role in enriching CaCO_3_ precipitation in a certain nutrient solution environment.

## Figures and Tables

**Table 1:** Basic physical and mechanical parameters of the SFS and the SC.

Soil	$\mathrm{Loose}\;\mathrm{Density}{g/\mathrm{cm}}^{3}$	$\mathrm{Bulk}\;\mathrm{Density}{g/\mathrm{cm}}^{3}$	$\mathrm{Dry}\;\mathrm{Density}{g/\mathrm{cm}}^{3}$	Void Ratio	Moisture Content %
SFS	1.58	1.80	1.62	1.14	35.12
SC	1.25	2.68	1.76	1.25	36.75

**Table 2:** Specimen grouting and preparation.

Group	Specimen	Ambient Temperature °C	Cementation Solution Concentration mol/L	Clay Content %
A	A1	15	1.0	10
	A2	20		
	A3	25		
	A4	30		
B	B1	20	0.5	
	B2		1.0	
	B3		1.5	
	B4		2.0	
C	C1		1.0	0
	C2			5
	C3			10
	C4			15

**Table 3:** Rate of void ratio, permeability, and CCC.

CC/%	12–24 h			24–48 h		
	Void Ratio 10^−3^/h	PC 10^−5^ cm/s/h	CCC 10^−2^ g/h	Void Ratio 10^−3^/h	PC 10^−5^ cm/s/h	CCC 10^−2^ g/h
0	−2.5	−3.33	3.67	−1.67	−2.92	7.81
5	−3.5	−5.01	9.75	−0.79	−1.25	4.01
10	−2.5	−9.17	6.00	−1.67	−2.08	2.07
15	−3.3	−7.52	5.55	−0.83	−3.75	0.88
